# The CRISP(Y) Future of Pediatric Soft Tissue Sarcomas

**DOI:** 10.3389/fchem.2020.00178

**Published:** 2020-03-13

**Authors:** Silvia Pomella, Rossella Rota

**Affiliations:** Department of Oncohematology, Bambino Gesù Children's Hospital, IRCCS, Rome, Italy

**Keywords:** CRISPR/Cas9, sarcoma, enhancers, chromosomal translocation, mice models

## Abstract

The RNA-guided clustered regularly interspaced palindromic repeats (CRISPR)/associated nuclease 9 (Cas9)-based genome editing technology has increasingly become a recognized method for translational research. In oncology, the ease and versatility of CRISPR/Cas9 has made it possible to obtain many results in the identification of new target genes and in unravel mechanisms of resistance to therapy. The majority of the studies have been made on adult tumors so far. In this mini review we present an overview on the major aspects of CRISPR/Cas9 technology with a focus on a group of rare pediatric malignancies, soft tissue sarcomas, on which this approach is having promising results.

## Introduction

**Figure 1:**
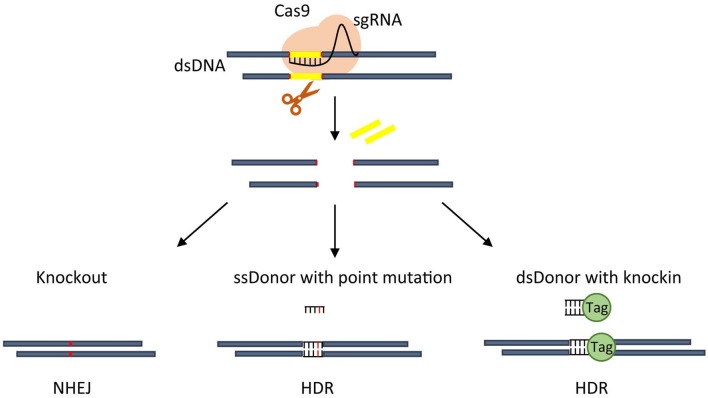
Overview of CRISPR-based approaches. An active CRISPR nuclease Cas9 is directed by sgRNA to make a cut on DNA that, through NHEJ repair, usually create a small indel thus allowing gene knockout (left). CRISPR-Cas9 guided cut, through HDR, can insert point mutation mediated by single strand (ss) donor with point mutation (middle), or generate an in frame knockin by using double strand (ds) donor carrying knockin sequence (right).

Starting from the works of Jinek et al. (2012, 2013) and Qi et al. (2013), the prokaryotic adaptive system for the defense against exogenous viruses called clustered regularly interspaced palindromic repeats (CRISPR) paired with the CRISPR-associated endonuclease 9 (Cas9) has been more and more recognized as a powerful and efficient tool for genome editing. The RNA-guided CRISPR/Cas9 consists of a small guide RNA (sgRNA) in complex with Cas9 and whose pairing with the target DNA induces a single Cas9-dependent double-strand breaks (DSBs) (as reviewed by Lino et al., 2018). The resulting editing includes deletions or insertions of specific sequences or changes of pre-existing DNA sequences in specific target regions of the genome of living cells (Figure 1). More recently, the technology has been enriched to label DNA regions (Banito et al., 2018), modulate endogenous gene expression (La Russa and Qi, 2015) or change the epigenetic status (Vojta et al., 2016). Compared to transcription activator-like effector nucleases (TALENs) and zinc-finger nucleases (ZFNs) technologies, CRISPR/Cas9 allows multiplexed analyses, is of rapid construction and easier to deliver, has a higher editing efficiency, even if off-target cleavages are more frequent (Gupta and Musunuru, 2014).

In the last decade, this approach has entered the basic and preclinical oncologic research on cancer cell survival, metastasis, and drug resistance.

In this minireview, we will give an overview of the CRISPR/Cas9 technology with a particular focus on its use to unravel tumorigenic mechanisms and identify pathways potentially targetable in a group of translocation-positive pediatric soft tissue sarcomas (STSs).

## Challenges of CRISPR/Cas9 Technology

The repair of Cas9-created blunt-ended DSBs can be responsible for undesired effects including translocations (Kosicki et al., 2018) and/or activation of p53 (Haapaniemi et al., 2018; Ihry et al., 2018). Moreover, it often produces a mutational reproducible profile correlated to the target sequence (Brinkman et al., 2018).

The DSBs repair mainly uses the non-homologous end joining (NHEJ) or homology-directed repair (HDR). NHEJ is the usually active pathway in human cells, works faster than HDR but is error-prone randomly giving rise to insertions or deletions. It is able to specifically knockout a gene by creating frameshift mutations or translocations, even if with low efficiency, disrupting the coding sequence or the gene regulatory regions (as reviewed by Wang et al., 2020). Conversely, HDR is slower and more precise, operates only during certain cell cycle phases but needs a double-stranded donor to guide the repair. It is useful to introduce specific sequences (knockin) or mutations into a target genomic region or efficiently produce chromosomal translocations (as reviewed by Vanoli and Jasin, 2017).

Cas9 is a very efficient endonuclease and nucleotide binding does not require a complete identical sequence potentially allowing the annealing of mismatched sequences. Therefore, cleavages outside the on-target sites can lead to off-target genomic modifications causing unintentional impairment of normal genes expression or activation of silent genes (Fu et al., 2013; Anderson et al., 2018). Prediction and detection of off-targets events is still a key challenge for the field. Online tools have been developed to predict off-targets sites based on the specificity of sgRNA sequence (Haeussler, 2019; Li et al., 2019). In addition, biased methods based on the experimental detection of predicted off-target sites or unbiased off-target analysis to discover unintended cleavages can be applied for detecting off-targets events (Haeussler, 2019; Li et al., 2019).

Moreover, to increase the precision of DSB repair, base editing (Rees and Liu, 2018) or the very recently primed editing (Anzalone et al., 2019) have been suggested as alternative technologies, which can operate with no requirement of donor DNA templates. Primed edits can induce all the 12 possible base-to-base conversions directly into the genomic DNA decreasing off-targets. However, additional studies are needed to exploit all its potential.

## CRISPR/Cas9 Technology to Unravel Oncogenic Drivers in Pediatric Soft Tissue Sarcomas

Pediatric cancers affect children and adolescents and, even if considered rare due to the low number of patients, they are the leading cause of death in the young population (as reviewed by Kattner et al., 2019). They are different from the adult ones in term of pathogenic mechanisms and therapeutic approaches including the long-term side effects of therapy that highly impact the life of cancer survivors (Forrest et al., 2018).

Differently from adult cancers, characterized by a number of mutations and chromosomal alterations that accumulate over time (Siegel et al., 2020), pediatric cancers have a low mutational burden and about 10% of all pediatric tumors showed no mutation at all (Gröbner et al., 2018). This feature makes it difficult to find altered factors targetable with current therapies (Shern et al., 2014; Tirode et al., 2014; Pishas and Lessnick, 2016). This evidence also suggests that epigenetic rather than purely genetic abnormalities are involved in the malignant process (Monje, 2018).

We work in a Children's Hospital and Research Institute and we are particularly interested in solid tumors of childhood, and more specifically in rhabdomyosarcoma (RMS).

RMS is a myogenic STS of childhood, which includes translocation-positive and -negative subgroups. The most frequent recurrent chromosomal translocation *t*_(2;13)_ in RMS results in the expression of the fusion oncoprotein PAX3-FOXO1, necessary for tumor cell survival (Gryder et al., 2017). Fusion-negative RMS are frequently RAS-mutated (Skapek et al., 2019). Despite currently available multimodal therapies, the prognosis for high risk patients with metastatic disease, including those harboring PAX3-FOXO1, remains dismal with a 5-year overall survival <30%, highlighting the need for novel therapeutic approaches (Missiaglia et al., 2012).

A number of STSs are dependent from fusion proteins that act as or in complexes with transcription factors (TFs) such as the afore-mentioned PAX3-FOXO1 in RMS, EWS-FLI1 in Ewing sarcomas (ES), an STS that affects also bones, and SS18-SSX in synovial sarcomas (SS) (Riggi et al., 2014; Gryder et al., 2017; McBride et al., 2018). These oncoproteins work in complexes of core regulatory (CR) TFs, specific for each type of tumor, regulating an oncogenic gene transcriptional program through binding to enhancer regions (Gryder et al., 2019a,b).

In addition, loss of specific gene components of the SWI/SNF chromatin-remodeling complex such as *SMARCB1* or *SMARCA4* characterizes rhabdoid sarcomas, another type of pediatric STS (Roberts et al., 2000; Lawrence et al., 2013). The SWI/SNF complex is considered a tumor suppressor and drives differentiation maintaining proper physiological enhancer functions (Alver et al., 2017; Wang et al., 2017). Overall, these TFs-dependent cancers can be considered “enhancers-driven” diseases, an aspect that has been unveiled only recently.

Unfortunately, TFs and nuclear DNA-bound complexes are difficult to target directly, and even more in the case of loss of function of a single gene. However, several new approaches are giving promising results (as reviewed by Bushweller, 2019). In this view, the identification of the bound enhancer regions and the composition and functioning of such transcriptional complexes has high relevance for the discovery of novel targetable molecules in pediatric STSs.

Enhancers are distally located short regions of non-coding DNA that “facilitate/enhance” the expression of a *cis*-encoded gene (Moreau et al., 1981). They act over a few to tens of kilobases, bear histone marks associated with open chromatin (H3K27ac, H3K4me1), are bound by cell-type specific TFs and then loop in 3D space to interact with their related promoters (Spitz and Furlong, 2012). In recent years, different methodologies have been applied to correctly identify, validate and characterize enhancers, among which (i) DNA sequence to map TFs binding motifs (ii) biochemical annotations to describe chromatin status (ChIP-seq, CUT&RUN, DNase-seq, MNase-seq, and ATAC-seq, DNA methylation, PRO-seq, RNA-seq), (iii) 3D conformation mapping to visualize enhancer-promoter communication (ChIA-PET, HiChIP, PLAC-seq, DNase Hi-C, 3C, 4C), (iv) massively parallel reporter assays (MPRAs) to test the functional activity of many candidate enhancers using barcodes (as reviewed by Gasperini et al., 2020). The emergent CRISPR/Cas9 technology is fully applied to perturb enhancer activity complementing the previous methodologies. CRISPR-based approaches are, indeed, used to: disrupt a TF binding site, delete the entire enhancer, engage a repressive or activating domain through a nuclease inactive dead (d)Cas9 (Qi et al., 2013) on a specific enhancer site to disrupt or activate its activity. CRISPR enables to specifically target short DNA sequences to precisely define the site necessary for TF transcriptional activity and the 3D fold looping.

In RMS we recently discovered CR TFs including PAX3-FOXO1 and the cell lineage type-specific master TFs MYOD and MYOG (Gryder et al., 2019a). We showed that these CR TFs co-regulate transcriptionally each other and activate the transcription of oncogenic genes in RMS by binding to SEs forming a tumor core regulatory circuitry (Gryder et al., 2019a). We were able to confirm the essential role of these CR TFs for cell survival and proliferation applying a CRISPR/Cas9 pooled library targeting each DNA-binding domain of all TFs expressed in one PAX3-FOXO1 RMS cell line, including the CR TFs, and comparing the results to those of all non-CR TFs (Gryder et al., 2019a).

Interestingly, EWS-FLI1 in ES binds to microsatellite chromatin regions at SEs to activate the transcription of target genes (Riggi et al., 2014). With the aim to identify mediators of EWS/FLI1 function, the component of chromatin-activating machinery BRD4 was pharmacologically inhibited in ES leading to the identification of PHF19, a protein associated with PRC2, as a novel EWS-FLI1-regulated gene (Gollavilli et al., 2018). Targeting of PHF19 with the CRISPR/Cas9 approach highlighted the contribution of this gene to the oncogenic program in ES.

In SS, the fusion oncoprotein SS18-SSX incorporates into SWI/SNF (BAF) complexes causing the eviction of the BAF47 tumor suppressor subunit, encoded by *SMARCB1* (Kadoch and Crabtree, 2013). A subsequent work of the Kadoch's group demonstrated that the incorporation of SS18-SSX into the BAF complexes hijacks them from enhancers to PRC2-controlled bivalent promoters activating these latter and blocking, in the meantime, the accessibility of distal (enhancer) sites (McBride et al., 2018). This phenomenon was sufficient to foster a tumorigenic gene signature. SS18-SSX depletion led to the retargeting of BAF complexes to enhancers sites only in cells in which *SMARCB1* has not been knocked out by CRISPR/Cas9, suggesting BAF47 as necessary for this step. Conversely, BAF47 appeared not needed for the proliferative arrest, as reported in rhabdoid tumors with loss of *SMARCB1* (as reviewed by Kohashi and Oda, 2017), observed after SS18-SSX depletion (McBride et al., 2018). These results indicate that the roles for SSX18-SSX knock down and BAF47 rescue are functionally decoupled in SS.

Genome-scale CRISPR-Cas9 gene-knockout screens alone or in combination with small drug screenings are becoming increasingly employed in the study of pediatric cancers (Aguirre et al., 2016; Oberlick et al., 2019).

Actually, these types of large-scale perturbational screening in tumor cell lines can unveil novel therapeutic vulnerabilities in genomes lacking mutations or genomic rearrangements, which can be useful for preclinical investigations.

Through this approach, Oberlick et al. discovered a dependency of rhabdoid cells on different receptor tyrosine kinases (RTKs) among which *PDGFRA* and *MET* (Oberlick et al., 2019). They demonstrated that these kinases are up-regulated following *SMARCB1* loss in cells lines and primary samples providing a proliferative advantage (Oberlick et al., 2019).

Stolte et al. (2018) used the data of their published CRISPR/Cas9 genome wide screening (Aguirre et al., 2016) to identify among others ES cell lines behaving as p53 wild-type, i.e., dependent on p53, better representing primary ESs genomic status conversely to the available p53m ES cell lines. Then, gene anti-correlated with the p53-dependency score such as *MDM2, MDM4, PPM1D*, and *USP7* were identified as necessary for cell survival of *TP53* wild-type ESs through pharmacological targeting with inhibitors or stapled peptides suggesting future clinical interventions.

Platforms for forward genetic screening using CRISPR/Cas9 have been used as a model to unravel mechanisms of drug actions and drug resistance in various cancer types. In addition, treatment of tumor cells with drugs and/or small molecules in combination with CRISPR/Cas9-induced specific deletions can reveal synthetic lethal interactions that can be potentially valuable for future treatments. It is known that tumor cells with defective DNA mismatch repair (dMMR) develop drug resistance due to mutations leading to drug-resistant alleles that can be identified by transcriptome or whole-exome sequencing (Han et al., 2017). Intriguingly, Povedano et al. (2019) exploited CRISPR/Cas9 genome editing to delete *MSH2*, a mismatch repair gene, in ES cells. Through this approach, they circumvent the paucity of mutations of this pediatric tumor inducing microsatellite instability and the emergence of hypermutated compound-resistant clones, which ultimately could facilitate the discovery of drug resistance mechanisms (Povedano et al., 2019).

Deletions of portions of a gene by CRISPR/Cas9 to study the contribution and requirement of specific domains for nuclear proteins has been applied to the histone demethylase KDM2B in SS (Banito et al., 2018). Banito et al. (2018) indeed, using this strategy discovered that the DNA binding domain of KDM2B, and not its enzymatic activity, is needed in SS cells to mediate the SS18-SSX binding to chromatin, which in turn leads to the de-repression of proliferative genes (Banito et al., 2018). Moreover, thanks to a tag inserted by CRISPR/Cas9 at the N-terminal of SS18-SSX, which is challenging to immunoprecipitate with usual antibodies, interactions between the fusion protein and almost all the components of the non-canonical Polycomb Repressor Complex (PRC) 1.1, among which KDM2B, were identified. Overall, a dependency of SS18-SSX on an epigenetic player like KDM2B has been thus unveiled.

Through CRISPR/Cas9 it has also been possible to model chromosomal translocations specific of ES (*EWS-FLI1*) and desmoplastic small round cell (DSRC) tumors (*EWS-WT1*) in HEK293 and human Mesenchymal cells (Torres et al., 2014; Spraggon et al., 2017) and RMS (*Pax3-Foxo1*) in murine myoblasts and mice *in vitro* (Lagutina et al., 2015).

To enhance the efficiency of rearrangements, the group of Ladanyi (Spraggon et al., 2017) combined CRISPR/Cas9 with HDR. The clear advantage of obtaining the expression of chromosomal translocations products is multiple because the role of specific fusion factors as drivers of the tumor can allow the discovery of the permissive cell context and can be exploited for drug testing.

**Figure 2:**
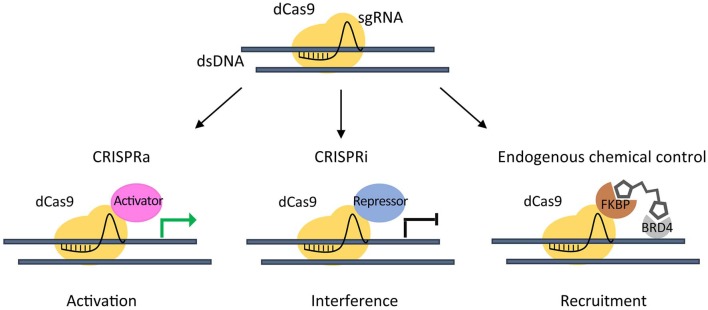
CRISPR/dCas9 chromatin engaging approaches. Inactive (dead) dCas9 is tethered to an activating domain (CRISPR activation- CRISPRa) that can potentially induce activation of a target gene (green arrow) (left), or to a repressive domain (CRISPR interference - CRISPRi) that can interfere gene expression (black arrow) (middle). A dCas9-FKBP fusion protein is used to recruit Chemical Epigenetic Modifiers (CEM) small molecules, in order to engage the endogenous BRD4 to a target gene (right).

The emerging need to study chromatin regulation in a more physiological context, led to the development of the inactive endonuclease dCas9 (Qi et al., 2013) fused to transcriptional transactivation domains of a specific factor. This complex is able to recruit chromatin effectors to a target gene by sgRNAs to repress (Gilbert et al., 2013) or activate its expression (Hilton et al., 2015) (Figure 2).

Taking advantage of a dCas9 fused to truncated FKBP protein that can recruit Chemical Epigenetic Modifiers (CEM) small molecules, we were able to engage the endogenous BRD4 to target super-enhancer (SE) elements thus inducing an at least 20-fold increase in the activation of the already expressed *MYOD1* gene in PAX3-FOXO1 RMS cells (Chiarella et al., 2020). This method enabled the activation of endogenous genes avoiding the need of exogenous transcriptional activators.

## *In vivo* Sarcoma Models Using CRISPR/Cas9

Genetically engineered mouse models (GEMM) of adult cancers have been created or used for the study of gene drivers by CRISPR/Cas9 (Heckl et al., 2014; Platt et al., 2014; Sanchez-Rivera et al., 2014; Chiou et al., 2015) or to generate actionable gene fusions such as EML4-ALK typical of lung cancers (Maddalo et al., 2014).

Albers et al. (2015) developed a lentiviral vector inducing the co-expression of *Cas9, H-Ras*^*G*12*V*^ and multiple sgRNAs against different genes such as *Trp53, Cdkn2a* and *Pten* (MuLE). By injecting MuLE vectors into the skeletal muscles of mice, they were able to show genetic cooperation between oncogenes and tumor-suppressor genes in generating models of undifferentiated sarcoma with pleomorphic (UPS) and fusion-negative RMS characteristics (Albers et al., 2015).

To obtain a fusion-positive model of RMS, a translocation of the murine *Pax3-Foxo1* has been produced in mice firstly using an engineering strategy to invert *Foxo1* orientation, which is in an opposite orientation to *Pax3*, in murine ES cells (Lagutina et al., 2015). Then the authors isolated and cultured myoblasts and fibroblasts (MEFs) from these *Foxo1*^*inv*+/+^ mice to virally deliver Cas9 and *sgRNAs in vitro* and produced a *t*_(1;3)_ translocation, correspondent to the human *t*_(2;13)_, and a functional Pax3-Foxo1 protein. Thus, these mice can be useful for future investigations on the tumorigenic steps of this chromosomal translocation (Lagutina et al., 2015).

Recently, a combined Cre-loxP and CRISPR/Cas9 approach in the production of GEMM of adult and pediatric sarcomas revealed the potentiality in generating UPS and malignant peripheral nerve sheath tumor (MPNST) sarcomas (Huang et al., 2017). UPS spontaneous tumors were generated in a Cre-loxP mouse model conditionally expressing oncogenic *Kras* and *Cas9* after an intramuscular injection of an adenoviral Cre-recombinase and an sgRNA for *Trp53* (the homologous of the human *TP53*). More interestingly, a single injection into the sciatic nerve of wild-type mice of an adenoviral vector co-expressing Cas9 and single sgRNAs individually targeting *neurofibromin 1* (*Nf1*) and *Trp53* induced the development of MPNSTs (Huang et al., 2017). These two types of sarcomas developed *in vivo* with a similar tumor onset, resembling their human counterparts, with similar characteristics of sarcomas obtained with the Cre-loxP system. Overall, the above CRISPR-based sarcoma models pave the way for future investigations by a growing number of researchers decreasing both the expenses and the time to obtain them.

## Concluding Remarks

Pediatric sarcomas are rare tumors molecularly highly heterogeneous and genetically diverse. The potentiality offered by the CRISPR/Cas9 has just begun to be exploited in this field and there is still much to study. In this “Gene Therapy Era” in which diseased people can be cured by CRISPR/Cas9-induced gene replacement, this technology has a lot to offer and new CRISPR approaches are continuously in development such as that using Cas13 nuclease able to cleave RNA molecules (Gulei et al., 2019). Several clinical studies based on CRISPR/Cas9 are underway for solid and hematological malignancies, infections (HIV and gastrointestinal), Sickle Cell Disease and Thalassemia (www.clinicaltrials.gov), which are expected to become ever more numerous.

In the study of pediatric STSs, the CRISPR/Cas9 can help in the understanding of mechanisms of malignancy development unveiling the cell(s) of origin of each subtype, which is still unknown for the majority of them, by manipulating normal cells/tissues and/or GEM models. In this regard, this technology, together or not with Cre-recombinase delivery *in vivo*, can be used to generate GEMM with mutations in multiple driver genes to obtain models of cancers bearing complex and heterogeneous genomic alterations.

Moreover, the huge effort in obtaining patient-derived models of rare cancers including pediatric STSs, can define a platform to combine CRISPR/Cas9 functional genomic screening with drug screenings to assist in the identification of novel targets in the “Precision Medicine” age.

It is however noteworthy that for preclinical studies aimed at identifying targetable genes, the combination of two techniques such as CRISPR/Cas9 and RNAi more robustly can confirm the results in the studied context. Indeed, the former induces gene deletions while the latter leads to suppression of gene expression, but both have specific off-target effects. In addition, in both cases tumor cells can show adaptation to long-term gene disruption/suppression, an effect that should be taken into account.

Oncological research and therapy have recently been revolutionized by the advent of immunotherapy giving hope even for forms of cancer considered incurable. A number of clinical trials are under evaluation for adoptive therapy including chimeric antigen receptor CAR-T cells in adult and pediatric sarcomas (www.clinicaltrials.gov). However, the majority of STSs show low if not a lack of T cells infiltration and inflammation and are so-called “cold tumors” (Bonaventura et al., 2019) and, consequently, not responsive to immunotherapeutic approaches requiring T cells homing. With respect to this latter aspect, the emerging data on the modulation of immune-checkpoints by CRISPR/Cas9 (Zhang et al., 2018; Afolabi et al., 2019) pave the way to a future of hope in switching the tumor microenvironment from “cold” to “hot” also in STSs.

## Author Contributions

RR conceived the paper and selected the literature. SP and RR wrote the paper, read, and approved the final manuscript.

### Conflict of Interest

The authors declare that the research was conducted in the absence of any commercial or financial relationships that could be construed as a potential conflict of interest.
